# Pilot Study on Nanofiltration Process for Surface Water Treatment and Optimization in Northern Jiangsu Region

**DOI:** 10.3390/membranes16040117

**Published:** 2026-03-27

**Authors:** Jiaming Jin, Sicheng He, Tao Zhang, Shengji Xia

**Affiliations:** 1 College of Environmental Science and Engineering, Tongji University,1239 Siping Road, Shanghai 200092, China2331362@tongji.edu.cn (S.H.);; 2 College of Construction Equipment, Zhejiang College of Construction, Hangzhou 310000, China; 3 Shanghai Municipal Engineering Design Institute<Group> Co., Ltd., Shanghai 200092, China

**Keywords:** nanofiltration, process optimization, pilot test

## Abstract

Nanofiltration (NF) is increasingly applied for advanced drinking water treatment, but achieving stable operation at high recovery rates remains challenging for surface waters with high scaling potential. This pilot study investigated the performance and optimization of a three-stage NF270 system (4:2:1 tapered array) for treating coagulated surface water in northern Jiangsu, China, aiming to identify sustainable operating conditions for high-recovery applications. The NF system was operated at recoveries of 80–90% with a feed flux of 20–23 LMH, and the effects of forward flushing frequency, acid dosing location, and concentrate recirculation on fouling behavior were evaluated. The NF270 membrane achieved consistent removal of organic matter (effluent chemical oxygen demand (COD_Mn_) < 0.5 mg/L), hardness (40–60% rejection), and alkalinity (~20% rejection), meeting Jiangsu Province drinking water standards. However, operation at 90% recovery resulted in rapid third-stage fouling, with permeate flow declining by >60% within 2.5 h. Osmotic pressure analysis (local concentrate osmotic pressure: 3.8–4.2 bar; net driving pressure: 0.8–2.2 bar) confirmed physical scaling rather than hydraulic limitation as the dominant mechanism. Stage-wise concentration factor calculations (CF_1_ = 1.6, CF_2_ = 2.9, CF_3_ = 4.4) revealed local Langelier Saturation Index (LSI) values of 1.8–2.2 in the third stage, identifying CaCO_3_ supersaturation as the primary scaling cause. Reducing recovery to 85% and flux to 20 LMH with 2 h forward flushing extended stable operation. Acid addition effectively mitigated scaling, but dosing location was critical: first-stage addition (pH 8.1 → 7.6) reduced third-stage LSI to 0.7–0.9 and stabilized performance, whereas third-stage addition (pH 8.0 → 7.3) inadvertently promoted Al(OH)_3_ precipitation from residual coagulant (feed Al: 0.07–0.11 mg/L). Concentrate recirculation (90% ratio) did not alleviate fouling. These findings demonstrate that for aluminum-rich coagulated surface waters, optimizing recovery, flushing frequency, and acid dosing location is essential for sustainable NF operation, and provide engineering guidance for full-scale applications.

## 1. Introduction

In current urban drinking water treatment plants, the main drinking water treatment process is the conventional treatment process, which includes traditional coagulation, sedimentation, filtration, disinfection and other processes. The main removal targets of the conventional treatment process are suspended solids, colloids, pathogenic microorganisms, etc., in the source water. After a century of development, the conventional drinking water process has become increasingly mature and has made important contributions to ensuring the health of human drinking water. However, the conventional treatment process still has some limitations, such as poor removal of micro-polluted organic matter, insignificant removal of hardness, and no obvious removal effect on disinfection by-products. Nanofiltration membranes exhibit excellent ability to retain pollutants while allowing water to pass through, and require lower pressure than reverse osmosis (RO) membranes. Advanced nanofiltration membrane water treatment technology can remove nitrates, arsenic, fluorides, heavy metals and pesticide residues from drinking water [1,2], while allowing beneficial minerals (such as calcium and magnesium) to pass through into the permeate, thus maintaining the mineral content essential for human health. Nanofiltration technology is characterized by good stability, low chemical consumption, and easy management and maintenance [3,4,5,6,7]. It is an effective method for preparing high-quality drinking water at present and has a good application prospect.

The nanofiltration process can achieve advanced removal of dissolved organic matter and some ions in water based on the effluent of the traditional water treatment process, and at the same time achieve good retention of residual microorganisms and viruses in water. It is a reliable water purification technology in the current drinking water process [8,9,10,11,12]. The nanofiltration process is a membrane system that connects a certain number of nanofiltration membrane elements in series and parallel in different connection forms, and is equipped with complete chemical dosing, flushing and chemical cleaning facilities. In a typical NF system design, the permeate recovery per 8-inch membrane element is limited to approximately 15% to maintain sufficient cross-flow velocity and minimize concentration polarization. Similarly, the recovery per stage is typically designed not to exceed 50% to avoid excessive scaling potential in the terminal elements [13,14]. To ensure a high product water volume, the NF system is usually designed as a multi-stage system. A booster pump is installed between the two stages to compensate for the pressure drop caused by the filtration process of the previous stage, thereby maintaining a certain product water flow rate. At the same time, the concentrated-water from the previous stage enters the membrane elements of the next stage for further filtration treatment, improving the recovery rate of the NF system. When the NF system can operate stably, increasing the recovery rate of the NF system can reduce the amount of wastewater discharge and improve the operation efficiency of the water plant.

The primary objectives of this pilot study were: (1) to evaluate the performance of a three-stage NF270-based system for treating coagulated surface water in northern Jiangsu, focusing on organic matter, hardness, and alkalinity removal; (2) to investigate the stability of the system at high recovery rates (85–90%) and identify the dominant fouling mechanisms through osmotic pressure analysis and stage-wise concentration factor calculations; (3) to optimize operating conditions—including forward flushing frequency, acid dosing location, and concentrate recirculation—to achieve stable long-term operation; and (4) to provide engineering recommendations for full-scale NF applications treating similar water sources. The results are compared with the literature data to contextualize the findings.

## 2. Materials and Methods

### 2.1. Influent Water Quality

The pilot test was carried out in a water plant in northern Jiangsu, with river water as the water source. The raw water was treated by the conventional treatment process (coagulation, sedimentation, filtration, disinfection) and ultrafiltration (UF) process of the water plant, and then used as the influent of the nanofiltration (NF) system. The UF process adopted a pressure-type membrane module (Sinno WTM500-P80, Beijing, China), with a nominal pore size of 0.1 μm and a designed operating flux of approximately 70 L/(m^2^·h). The test was conducted in January 2023, and the water quality indicators during this period were as follows: temperature 5–10 °C, electrical conductivity 482–708 μS/cm, COD_Mn_ 0.96–1.4 mg/L, total alkalinity 118.2 mg/L, and total hardness 154–171 mg/L. During the operation, the influent temperature, electrical conductivity, COD_Mn_ and other indicators of the NF system were monitored daily. Water quality parameters were analyzed according to standard methods (GB/T 5750-2023) [15]. Turbidity was measured using a turbidimeter (Hach 2100Q, Loveland, CO, USA), pH by a pH meter (Mettler Toledo FE28, Greifensee, Switzerland), and temperature by in-line sensors. COD_Mn_ was determined by acidic potassium permanganate titration, UV_254_ by spectrophotometry (Shimadzu UV-2600, Kyoto, Japan), and DOC/TN by a total organic carbon (TOC)/TN analyzer (Shimadzu TOC-L CPH, Kyoto, Japan). Total phosphorus was measured by ammonium molybdate spectrophotometry after alkaline persulfate digestion. Total alkalinity and hardness were determined by hydrochloric acid and ethylenediaminetetraacetic acid (EDTA) titration, respectively. Anions (F^−^, Cl^−^, NO_3_^−^-N, SO_4_^2−^) were analyzed by ion chromatography (Dionex ICS-5000, Thermo Scientific, Sunnyvale, CA, USA), and metal ions (Al, Ca, Fe) by inductively coupled-plasma mass spectrometry (ICP-MS, Agilent 7900, Santa Clara, CA, USA). Total dissolved solids (TDS) was determined gravimetrically.

### 2.2. Experimental Equipment

**Figure 1:**
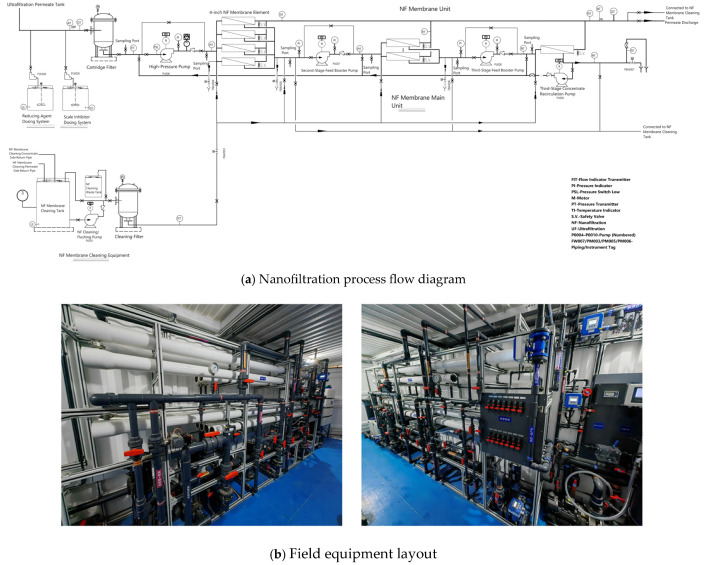
Schematic diagram of the nanofiltration pilot system: (**a**) process flow, (**b**) field equipment layout.

The NF membrane system of the pilot equipment (Figure 1) was arranged in a one-stage, three-section mode. The second and third sections could be equipped with concentrated-water circulation to make part of the concentrated-water return to the front end. The ratio of the sections was 4:2:1, that is, 4 membrane housings in the first section, 2 membrane housings in the second section, and 1 membrane housing in the third section. Each membrane housing contained 7 membrane elements, totaling 49 membrane elements.

Based on this 4:2:1 configuration and the designed system recovery of 80–90%, the stage-wise recovery rates were estimated. Assuming uniform permeate flux across all membrane elements, the first section (with 4 housings) contributes approximately 57% of the total permeate, the second section (2 housings) contributes 29%, and the third section (1 housing) contributes 14%. At 90% system recovery, cumulative recoveries are 51%, 77%, and 90%, respectively. Thus, the third-stage feed is concentrated by a factor of 4.4 (CF = 1/(1−0.77)), which explains why fouling consistently initiates in the third section—this is where the highest solute concentrations and greatest scaling potential occur. Due to the fact that the length of the 7-core direct connection exceeded the maximum size of the container, a complete membrane housing in each section was composed of a long membrane housing filled with 4 membrane elements and a short membrane housing filled with 3 membrane elements connected up and down. The NF process used 4-inch NF membrane elements, with an effective membrane area of 7.6 m^2^ per NF membrane element and a total membrane area of 372.4 m^2^. The test was designed with an influent flow rate of 10 m^3^/h, and the water purification effect and operation stability of the NF system were investigated under a recovery rate of 80–90%.

### 2.3. Experimental Scheme

The NF270 membrane (DuPont, Edina, MN, USA) was selected for this study due to its widespread application in surface water treatment for potable production [8,12]. This membrane is a polyamide thin-film composite membrane with a molecular-weight cut-off (MWCO) of approximately 200–400 Da, characterized by high permeability and moderate rejection of monovalent ions while effectively retaining divalent ions and organic matter [3,5]. Its fouling behavior and cleaning strategies have been extensively documented, providing a benchmark for comparison with the results obtained in this study. First, under the condition of a system recovery rate of 90%, the operation stability and product water quality of the NF system under a membrane flux of 20 LMH-23 LMH were investigated. Then, different operating conditions were adopted to optimize the NF system under a high recovery rate. The optimization tests included a high-frequency forward flushing test, a three-stage acid addition test and a three-stage concentrated-water reflux test. In this test, each stage of the NF system was forward-flushed separately; that is, the second stage was flushed first, then the third stage, and, finally, the first stage. The forward flushing parameters of the NF system are shown in Table 1.

## 3. Results and Discussion

### 3.1. Operational Stability of Nanofiltration System Under High Recovery Rate

**Figure 2:**
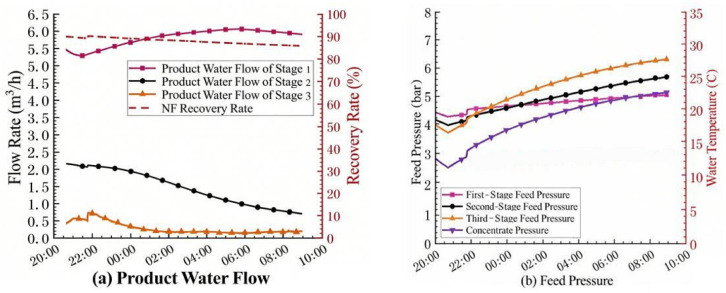
Trend of flow rate and pressure changes in nanofiltration water production: (**a**) flow rate change, (**b**) pressure change.

Previous studies have typically reported stable NF operation at recoveries of 75–85% for surface water treatment, depending on feed water quality and antiscalant efficiency [8,10,11]. However, as noted by Van der Bruggen et al. [16], achieving high recovery without accelerated fouling remains a key challenge for NF applications, often requiring additional scale control measures such as acid addition or optimized flushing. At present, domestic drinking water NF advanced treatment cases can basically achieve stable operation at an 85% recovery rate. Increasing the system recovery rate offers dual benefits: it maximizes permeate production for a given feed flow rate and reduces the volume of concentrated wastewater requiring subsequent treatment and disposal. Therefore, this pilot test first investigated the operational effect of the NF system under a high recovery rate. At the beginning of the test, the recovery rate of the NF system was set to 90%, the operating flux to 23 LMH, and the inter-stage pressure boost to approximately 1 bar. The antiscalant was D00 with a dosage of 3.0 ppm, and the reducing agent was sodium bisulfite with a dosage of 2.0 ppm. The test results are shown in Figure 2.

It can be seen from Figure 2a that after 2.5 h of operation, the product water flow rate of the third section of the NF system decreased by more than 60%. In Figure 2b, the operating pressure also showed an upward trend, and then the product water flow rate of the second section also began to decrease. This indicates that under high recovery rate, membrane fouling occurred in the third section of the NF system shortly after operation. With the extension of operation time, the fouling degree of the NF membrane elements in the second section gradually increased, and the product water flow rate began to decrease. At the same time, the pressure increase caused by membrane fouling in the third section was also transmitted to the second and first sections, resulting in a continuous increase in the influent pressure of the system.

To further diagnose the cause of the sharp performance decline, the osmotic pressure of the concentrate stream was estimated to distinguish between hydraulic limitations and chemical scaling effects. The osmotic pressure (π) can be approximated using the van ‘t Hoff equation: π = iCRT, where i is the van ‘t Hoff factor, C is the molar concentration of ions, R is the ideal gas constant (0.08314 L·bar·mol^−1^·K^−1^), and T is the absolute temperature (K).

Based on the feed water ion composition (Table 2), the total ionic concentration of the NF feed was calculated to be approximately 10 mmol/L as NaCl equivalent. At a recovery rate of 90% with a 4:2:1 staged array, the concentration factor in the third-stage feed was estimated to be 4.4, and the concentration polarization factor near the membrane surface was approximately 2.0. Thus, the local concentration at the membrane surface in the third stage was estimated to be 0.08–0.09 mol/L, corresponding to an osmotic pressure of 3.8–4.2 bar under the operating temperature of 5–10 °C.

The initial net driving pressure (NDP) for the third stage, defined as the transmembrane pressure (TMP, approximately 5–6 bar at the third-stage inlet) minus the osmotic pressure difference (Δπ) between the feed and permeate (assuming permeate osmotic pressure ≈ 0), was calculated to be approximately 0.8–2.2 bar. As shown in Figure 2, the permeate flux in the third stage declined by more than 60% within 2.5 h, while the applied pressure only increased marginally. If the flux decline were primarily due to increased osmotic pressure from concentration polarization, the inter-stage booster pump would compensate to maintain the NDP. However, the sustained decrease in permeate flow suggests a significant increase in membrane hydraulic resistance. This indicates that the dominant fouling mechanism is not simply an osmotic pressure limitation but the formation of a physical fouling/scaling layer on the membrane surface, which drastically reduces membrane permeability. To further identify the nature of this physical fouling layer, the stage-wise concentration factors and mineral supersaturation in the third stage were estimated. The third stage was concentrated to about 4.4 times the original feed concentration, while the local concentration at the membrane surface, accounting for concentration polarization, reached 7–9 times the feed concentration [17].

Using these concentration factors and the feed water ion composition from Table 2, the saturation indices for major sparingly soluble salts in the third stage concentrate were estimated. The results showed that the Langelier Saturation Index (LSI) for CaCO_3_ in the third-stage bulk concentrate reached 1.6, but the local LSI at the membrane surface was estimated to be 1.8–2.2, far exceeding the scaling threshold (LSI > 0). The saturation index (SI) for CaSO_4_ was calculated to be 0.6–0.8, below the gypsum precipitation threshold (SI > 1.0), indicating that calcium carbonate scaling was the primary risk [18].

These analyses confirm that the rapid fouling observed in the third stage was driven by severe CaCO_3_ supersaturation resulting from the high local concentration factors. This conclusion is consistent with the effective performance recovery observed after acid cleaning, which readily dissolves carbonate scales.

**Figure 3:**
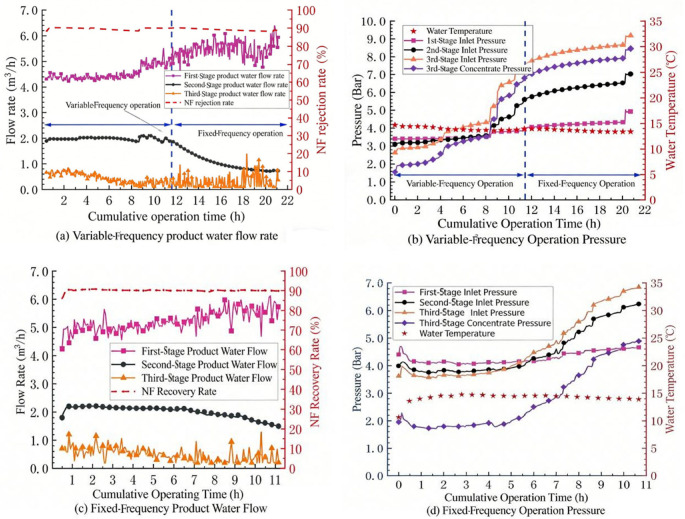
Trend of nanofiltration permeate flow rate and operating pressure in each section: (**a**) Variable-Frequency product water flow rate, (**b**) Variable-Frequency operation pressure; (**c**) Fixed-Frequency product water flow rate; (**d**) Fixed-Frequency operation pressure.

To restore the performance of the NF membrane, offline and online acidic chemical cleaning were performed on the NF membrane elements in the second and third sections. The results showed that acidic chemical cleaning had a good cleaning effect on the NF membrane. After acidic cleaning, the product water flow rates of the second and third sections of the NF system basically returned to the state before fouling. To maintain the normal operation of the system, it was decided to reduce the flux of the NF system to 21 LMH to reduce the concentration of pollutants in the concentrated-water at the end section of the system. The test results are shown in Figure 3.

After reducing the membrane flux, membrane fouling still occurred in the third section of the NF system: under this operating condition, the product water volume of the third section of the NF system began to decrease significantly after 4 h of operation; after 6 h, the product water flow rate of the third section was less than 0.5 m^3^/h, with a decrease of more than 30%. The product water flow rate of the second section also began to decrease after 6 h. Increasing the frequency of the booster pumps in the second and third sections (frequency conversion) to increase the pressure on the influent side of the membrane did not significantly increase the product water flow rate of the third section. In the later stage, an attempt was made to repeat the test under the condition of controlling the frequency of each water supply pump to be constant (fixed frequency), as shown in Figure 3c,d, but the product water flow rate of the third section still continued to decrease. This sequential fouling pattern is characteristic of a concentration-driven scaling mechanism in tapered arrays [19]. In the 4:2:1 configuration, the third stage receives the most-concentrated feed (CF ≈ 4.4), where the local LSI at the membrane surface reaches 1.8–2.2—well above the threshold for CaCO_3_ precipitation. The induction time for heterogeneous nucleation on the membrane surface under these supersaturation conditions is typically 2–4 h [20], consistent with the observed onset of flux decline. Once nucleation occurs, crystal growth rapidly increases hydraulic resistance, leading to the accelerated decline seen after 6 h. The ineffectiveness of pump frequency adjustment (Figure 3c,d) further supports this interpretation: increasing applied pressure cannot overcome the additional resistance from a continuous scale layer, and may even compress the foulant layer, exacerbating flux loss [21]. This mechanism contrasts with osmotic pressure-limited behavior, where flux would respond to pressure increases. It can be concluded that under this operating condition and chemical dosage, it is difficult for the NF system to operate stably.

The above research results show that when the raw water is the effluent from the carbon filter of Chengdong Water Plant and the dosage of D00 antiscalant is 3.0 ppm, it is difficult for the NF system to achieve stable operation at a 90% recovery rate. The possible reasons for the unstable operation of the NF system are as follows:

First, the high concentration of scale-forming ions (particularly Ca^2+^ and HCO_3_^−^) in the feed, combined with the stage-wise concentration factor of 4.4 in the third stage, resulted in local LSI values of 1.8–2.2, far exceeding the threshold for CaCO_3_ precipitation.

Second, the setting of NF operating conditions is not optimized: compared with the NF water plant using Yangtze River water as the water source, the TDS content in the raw water of the filter of this water plant is higher, and the pollutant concentration in the third section of the NF system will be higher, which is more obvious under a high recovery rate. It is necessary to adjust the operating conditions, such as increasing the NF flushing cycle, adding acid to adjust pH, and using three-stage concentrated-water reflux.

Therefore, the subsequent tests will solve the problem from the aspects of NF water purification effect and NF process adjustment and optimization.

### 3.2. Nanofiltration Water Purification Effect

Water samples were collected from the NF influent, concentrate, and permeate streams for comprehensive water quality analysis. The detection data are shown in Table 2. The ions in the water that may cause membrane fouling are mainly calcium, silicon, etc. The Langelier Saturation Index (LSI) of the NF concentrated-water was calculated to be 1.6 based on the bulk concentrate composition. However, as discussed in Section 3.1, this global LSI value underestimates the actual scaling potential at the membrane surface. Considering the stage-wise concentration factors (CF ≈ 4.4 for the third-stage feed and up to 7–9 at the membrane surface due to concentration polarization), the local LSI in the third stage was estimated to be 1.8–2.2, indicating a severe CaCO_3_ scaling risk. The saturation index (SI) for CaSO_4_ was calculated to be 0.6–0.8, suggesting that gypsum scaling was less likely under these conditions. The presence of aluminum (0.07–0.11 mg/L in the feed) may also contribute to fouling through the formation of aluminum silicates or Al(OH)_3_ precipitates at elevated concentrations [22,23]. According to the monthly sampling inspection results of the effluent from the carbon filter of the water plant, the aluminum content is basically maintained between 0.07 and 0.11 mg/L. Aluminum may change the solubility of SiO_2_ by forming metal silicates, and it can also combine with cationic flocculants to cause membrane fouling. In the subsequent tests, the problem of rapid fouling of the NF system was solved by adjusting the NF operating conditions and optimizing the selection of antiscalant.

**Figure 4:**
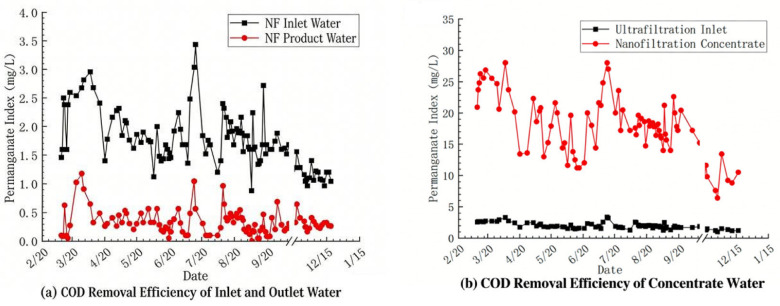
Effect of nanofiltration system on COD_Mn_ removal: (**a**) Inlet and Oulet Water, (**b**) Concentrate Water.

**Figure 5:**
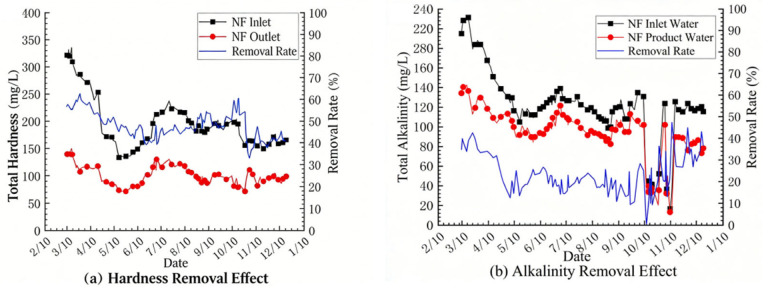
Effect of nanofiltration system on hardness and alkalinity: (**a**) Hardness, (**b**) Alkalinity.

The main purpose of this project is to investigate the removal effects of the NF system on organic matter, hardness and alkalinity in water. Figure 4 and Figure 5 show the removal efficiencies of selected pollutants by the nanofiltration system, as experimentally recorded from 2022 to 2023. It can be seen from Figure 4a that during the entire test process (excluding the lime softening period), the permanganate index of the product water of the NF system changed in the same trend as the influent value fluctuated. The influent COD_Mn_ of the NF system was basically less than 3.0 mg/L, and the product water COD_Mn_ was basically less than 0.5 mg/L, with a maximum of no more than 1.3 mg/L, which meets the standard requirement of 1.5 mg/L for the conventional process + advanced treatment process in Jiangsu Province. The NF270 NF membrane has an obvious removal effect on organic matter. The COD_Mn_ of the NF concentrated-water decreased with the decrease in COD_Mn_ in the influent, with a maximum concentration of no more than 30 mg/L. During the entire test cycle, as shown in Figure 5a, the removal rate of the NF270 NF membrane system for hardness was between 40 and 60%. The fluctuation trend of the hardness removal effect corresponds to the chemical cleaning frequency, and the removal rate of the NF system for alkalinity was basically maintained at around 20%. The observed removal rates for hardness (40–60%) and alkalinity (~20%) are comparable to those reported for NF270 membranes treating similar surface waters. For example, Song et al. [24] reported hardness rejection of 45–55% and alkalinity rejection of 15–25% for NF270 treating a river water source in China. The consistent removal of organic matter (COD_Mn_ < 0.5 mg/L) is also in agreement with previous studies [25,26], confirming the suitability of NF270 for producing high-quality drinking water. Comparing the conventional water quality indicators of the NF system with the national and Jiangsu provincial drinking water quality standards, the product water quality of the NF system is significantly better than the standard requirements.

### 3.3. Nanofiltration Working-Condition Adjustment Tests

**Figure 6:**
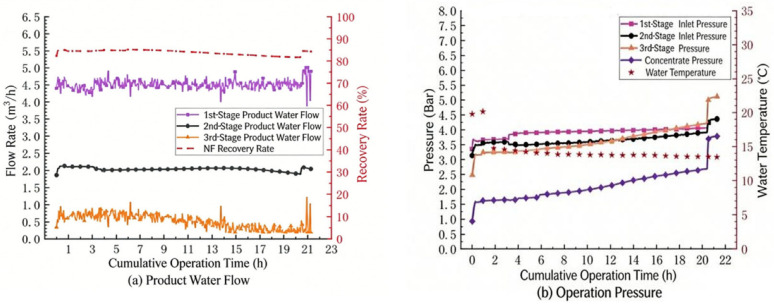
Trend of nanofiltration operating parameters (recovery rate 85%): (**a**) Product Water Flow, (**b**) Operation Pressure.

The operating-condition adjustment tests aim to improve the operational stability of the nanofiltration system through three methods: shortening the forward flushing cycle, conducting acid pretreatment, and increasing the recycling of the concentrated-water in the third stage. Before shortening the forward flushing cycle, the recovery rate of the nanofiltration system was reduced to 85%, and the membrane flux was decreased to 20 LMH, so as to reduce the concentration multiple of the concentrated-water at the end of the system and lower the possibility of membrane fouling caused by water quality factors. The test results are shown in Figure 6.

It can be seen from Figure 6a that after the recovery rate was reduced, the operation of the nanofiltration system was improved. During a one-day operation, there was no significant decrease in the water production flow rate of the second stage of the system. However, the water production flow rate of the third stage of the system began to decline after 10 h of operation. By 16 h, the water production flow rate of the third stage of the system had dropped from 0.8 m^3^/h at the beginning to 0.4 m^3^/h. As can be seen from Figure 6b, the influent pressures of the three stages had been on an upward trend throughout the operation cycle, which also indicated that under this working condition, the operation effect of the membranes in the third stage of the nanofiltration system was still not satisfactory and other working conditions still needed to be optimized simultaneously.

#### 3.3.1. High-Frequency Forward Flushing Test

**Figure 7:**
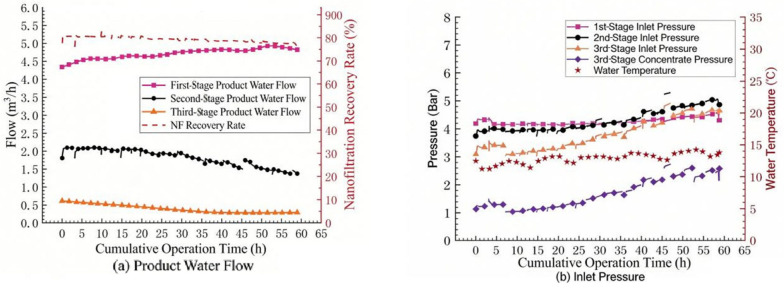
Trend of operating parameters for nanofiltration (high-frequency positive pulse): (**a**) Product Water Flow, (**b**) Inlet Pressure.

Considering the existence of membrane fouling in the nanofiltration system, an attempt was made to set the forward flushing cycle of the nanofiltration system to once every 2 h, and the effect of inhibiting the accumulation of membrane fouling was observed. The recovery rate of the system was still set at 85%, and the flux was 20 LMH. The test results are shown in Figure 7.

It can be seen from the figure that when the frequency of forward flushing is increased, the operation of the third stage of nanofiltration has been improved. The time point at which the water production flow rate drops by 10% has been extended to 24 h. However, it is also found that subsequently, the water production in the second and third stages still decreased simultaneously. As the operation time prolongs, the recovery effect of flushing becomes worse and worse. Even after the forward flushing is completed, the phenomenon of the water production flow rate slightly decreasing still occurs. This indicates that increasing forward flushing frequency alone was insufficient to completely mitigate membrane fouling under these operating conditions, suggesting that additional scale control measures are necessary for sustained long-term operation.

#### 3.3.2. Three-Stage Acid Addition Test

**Figure 8:**
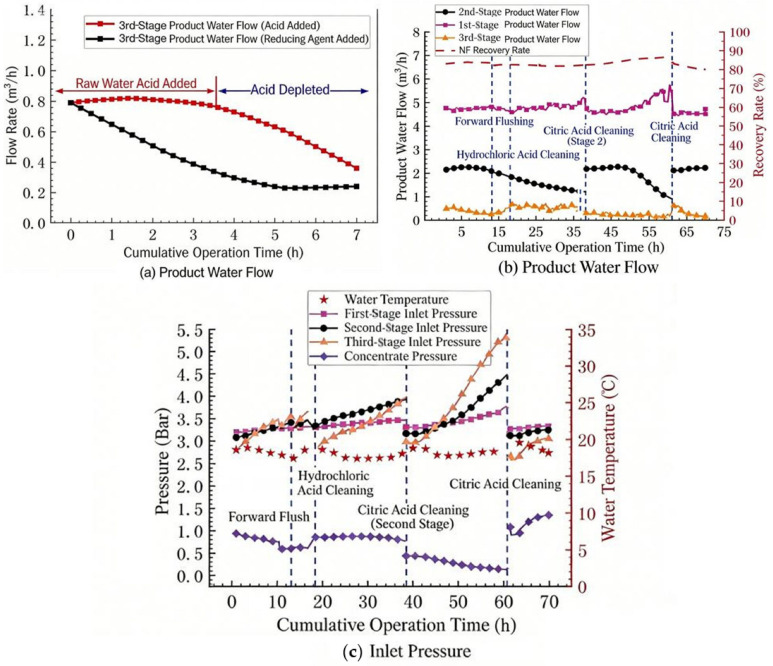
Trend of nanofiltration operating parameters (acid addition): (**a**) Permeate flow rate with acid/reducing agent dosing, (**b**) Permeate flow rate during acid cleaning, (**c**) Operating pressure during acid cleaning.

The change in the influent pH will affect the formation rate of membrane fouling. A decrease in pH can increase the solubility of some sparingly soluble substances in the concentrated water. During the pilot test, hydrochloric acid was added to the influent of the nanofiltration system through the influent pipeline of the nanofiltration, and the dosing point was located on the influent pipe of the cartridge filter. After adding hydrochloric acid, the pH of the second-stage concentrated water dropped from 8.1 to 7.6, and the Langelier index of the third-stage concentrated water decreased to 0.9, which also ensured that the risk of carbonate scaling was further reduced. The test results are shown in Figure 8a. It can be seen that adding hydrochloric acid before the influent of the first stage of the nanofiltration system can reduce the membrane fouling in the third stage and significantly delay the downward trend of the water production flow rate in the three stages of the nanofiltration system.

Although adding acid is effective in inhibiting membrane fouling, a relatively large amount of hydrochloric acid is consumed when added at the front end. Since membrane fouling mainly occurs in the third stage of the system, an attempt was made to add hydrochloric acid to the concentrated water in the second stage of the nanofiltration system (that is, the influent of the third stage) to reduce the pH of the influent of the third stage. After adjusting the pH of the influent of the third stage from 8.0 to 7.3 by adding acid, the variation trends of the water production flow rate and pressure of the nanofiltration system are shown in Figure 8b,c respectively. It can be seen that when acid is added before the influent of the third stage, the water production volume in the third stage of the nanofiltration system begins to decline significantly. After cleaning with citric acid and repeating the experiment, the same situation still occurs. The content of aluminum ions in the cleaning solution was relatively high (5.3 mg/L), suggesting that aluminum precipitation played a key role in the observed fouling.

To understand this phenomenon, a brief thermodynamic analysis of aluminum speciation as a function of pH was conducted. In aqueous systems, aluminum hydrolysis produces various species including Al^3+^, Al(OH)^2+^, Al(OH)_2_^+^, and amorphous Al(OH)_3_ precipitates, with the dominant species being determined by pH [22]. The minimum solubility of amorphous Al(OH)_3_ occurs in the pH range of 6.5–7.5, where the concentration of dissolved aluminum can be as low as 0.01 mg/L. At pH values below 6.0 or above 8.0, aluminum solubility increases significantly due to the formation of Al^3+^ or aluminate ions [Al(OH)_4_^−^], respectively [23].

In this study, the water plant uses polyaluminum chloride as a coagulant, resulting in residual aluminum in the feed (0.07–0.11 mg/L). When hydrochloric acid was added directly to the third-stage influent, the local pH was reduced from approximately 8.0 to 7.3. Although this pH reduction was intended to decrease CaCO_3_ supersaturation, it inadvertently shifted the feed into a pH range where aluminum solubility is minimized, promoting the precipitation of amorphous Al(OH)_3_. Under the concentrated conditions in the third stage (CF ≈ 4.4), the local aluminum concentration at the membrane surface could reach 0.3–0.5 mg/L, further increasing the precipitation potential. This explains the rapid fouling observed in Figure 8b and the high aluminum content detected in the cleaning solution.

In contrast, when acid was added to the first-stage influent, several factors mitigated aluminum fouling: (1) the acid addition point was upstream of the cartridge filter, allowing some precipitated particles to be intercepted; (2) the lower concentration factor in the first stage (CF ≈ 1.6) resulted in lower local aluminum concentrations; and (3) the pH adjustment occurred before significant concentration, potentially allowing aluminum precipitates to form under less severe conditions. This comparison highlights the importance of acid addition location when aluminum is present in the feed water.

#### 3.3.3. Three-Stage Concentrated-Water Circulation Test

Since it was always the membrane elements in the third stage of the nanofiltration system that got polluted first each time, turning on the concentrated-water circulation pump in the third stage of the nanofiltration system was considered. This would make part of the concentrated water in the third stage, after being pressurized by the circulation pump, mix with the concentrated water in the second stage and then enter the third stage together. In this way, the influent flow rate of the third stage could be increased, making the flow velocity on the surface of the membrane elements greater, so as to achieve the purpose of scouring the membrane surface and reducing membrane fouling.

**Figure 9:**
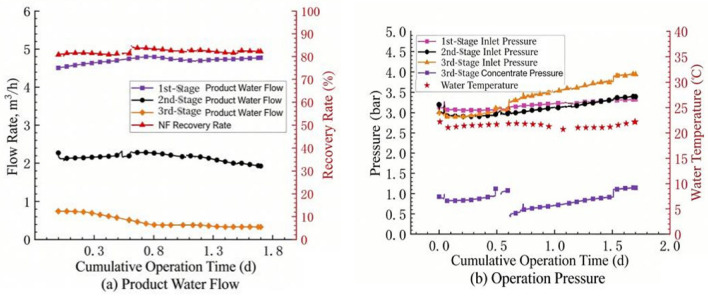
Trend of nanofiltration operating parameters (three-stage brine cycle): (**a**) Product Water Flow, (**b**) Operation Pressure.

Under the working condition where the concentrated-water circulation was not started, the influent flow rate of the third stage was 2.7 m^3^/h. After starting the concentrated-water circulation pump of the third stage, the flow rate of the concentrated-water circulation pump was 0.85 m^3^/h, the circulation ratio was 90%, and the concentrated-water discharge volume was 1.4 m^3^/h. At this time, the influent flow rate of the third stage was 3.03 m^3^/h, and the test results are shown in Figure 9. After starting the concentrated-water circulation pump of the third stage, the produced water flow rate of the third stage still showed an obvious downward trend, and the influent pressure kept rising, indicating that starting the concentrated-water reflux in the third stage could not effectively alleviate the nanofiltration membrane fouling situation under this working condition. After adjusting the typical operating conditions, the nanofiltration system still had the problem of unstable operation, and it might be caused by the externally added chemicals that led to a rapid decline in the membrane flux of the nanofiltration system [27,28].

## 4. Conclusions

This experiment systematically investigated the operating effects under various operating conditions of the nanofiltration process and the water purification efficiency of nanofiltration. The main conclusions are as follows:

(1) Operation at 90% recovery resulted in rapid CaCO_3_ scaling in the third stage within 2.5 h, despite antiscalant dosing (Section 3.1). Reducing the recovery to 85% with a flux of 20 LMH and forward flushing every 2 h extended stable operation, with the third-stage permeate flow declining from 0.8 m^3^/h to 0.4 m^3^/h after 16 h (Section 3.3). This demonstrates that for the feed water studied (Table 2), 85% represents the maximum sustainable recovery under the tested conditions without additional chemical intervention.

(2) The NF270 membrane exhibited consistent removal performance throughout the study: hardness rejection of 40–60%, alkalinity rejection of approximately 20%, and effluent COD_Mn_ consistently below 0.5 mg/L. These values meet Jiangsu Province standards for advanced treatment processes and are comparable to literature reports for similar applications [19]. The concentrated-water COD_Mn_ reached up to 30 mg/L, indicating effective organic matter accumulation and the need for concentrate management in full-scale applications.

(3) Acidic cleaning effectively restored membrane performance after CaCO_3_ scaling, with permeate flow rates returning to pre-fouling levels. However, when fouling was exacerbated by aluminum precipitation (as observed during third-stage acid addition), citric acid cleaning alone was insufficient to fully recover performance, and elevated aluminum concentrations (5.3 mg/L) were detected in the cleaning solution. This indicates that for feed waters containing residual coagulant, cleaning strategies may need to target both carbonate scales and aluminum-based foulants.

(4) Acid addition effectively mitigated CaCO_3_ scaling, but the dosing location critically influenced outcomes. First-stage acid addition (pH reduction from 8.1 to 7.6) reduced third-stage LSI from 1.8–2.2 to 0.7–0.9 and stabilized operation, whereas third-stage addition (pH 8.0 → 7.3) inadvertently promoted Al(OH)_3_ precipitation due to residual aluminum (0.07–0.11 mg/L) from coagulation. This highlights the importance of considering aluminum speciation when designing acid dosing strategies for NF systems treating coagulated surface waters.

## Figures and Tables

**Table 1:** Forward flushing parameters of nanofiltration system.

	The First Stage	The Second Stage	The Third Stage
flushing flow rate	9.0 m^3^/h	6.0 m^3^/h	3.0 m^3^/h
flushing time	3 min	3 min	3 min

**Table 2:** Water quality data of nanofiltration system.

Testing Item	Unit	Nanofiltration Influent	Nanofiltration Concentrated Water	Nanofiltration Permeate
Turbidity	NTU	<0.05	2.6	<0.05
Conductivity	μS/cm	1070	2920	678
TDS	mg/L	709	1930	455
pH	-	7.8	7.9	7.7
COD_Mn_	mg/L	2.8	14.30	0.50
COD_Cr_	mg/L	21	59	<4
DOC	mg/L	3.2	19.8	0.5
Total alkalinity	mg/L	196	535	139
NH_3_- N	mg/L	0.03	0.06	-
TN	mg/L	3.53	3.39	-
Chloride	mg/L	109	180	115
Bicarbonate radical	mg/L	239	652	169
sulfate	mg/L	152	1180	2
Sodium	mg/L	78.3	169	63.1
Magnesium	mg/L	31	150	9.77
Calcium	mg/L	87	335	38.9
Iron	mg/L	<0.02	<0.02	-
Silicon	mg/L	1.2	1.6	-

## Data Availability

The original contributions presented in this study are included in the article. Further inquiries can be directed to the corresponding author.
